# Is individualism-collectivism associated with self-control? Evidence from Chinese and U.S. samples

**DOI:** 10.1371/journal.pone.0208541

**Published:** 2018-12-19

**Authors:** Jian-Bin Li, Alexander T. Vazsonyi, Kai Dou

**Affiliations:** 1 Department of Early Childhood Education, The Education University of Hong Kong, Hong Kong; 2 Department of Family Sciences, University of Kentucky, Lexington, KY, United States of America; 3 Department of Psychology and Research Center of Adolescent Psychology and Behavior, School of Education, Guangzhou University, Guangzhou, P.R. China; Russian Academy of Medical Sciences, RUSSIAN FEDERATION

## Abstract

Self-control plays an important role in human’s daily life. In the recent two decades, scholars have exerted tremendous effort to examine the etiologies of the individual differences in self-control. Among numerous predictors of self-control, the role of culture has been relatively overlooked. In this study, the influences of cultural orientation on self-control were examined based on the collectivism-individualism framework using both self-report and behavioral task to assess self-control. A convenience sample of 542 Chinese and 446 U.S. undergraduates participated in the research. They were invited to fill out self-report questionnaires reporting their levels of attitudinal self-control and individualistic-collectivistic orientation after completing a computer-based Stroop task. Results of hierarchical regression models showed that Chinese participants reported less attitudinal self-control but had higher behavioral self-control than their U.S. counterparts. Moreover, individual-level individualism and collectivism was negatively and positively related to attitudinal self-control in both countries, respectively. Individual-level collectivism was significantly related to better behavioral self-control, but no significant results were found for the relationship between individual-level individualism and behavioral self-control. In sum, individualism and collectivism have some influences on individual differences in self-control. Implications for future research were discussed.

## Introduction

Self-control refers to one’s ability to change thoughts, emotions, and impulses to follow social norms, personal values, and to support the pursuit of long-terms goals [1]. Successful self-control brings immense benefit for personal and social thriving whereas self-control failure is costly in many aspects, such as leading to poor adjustment, criminal and deviant behavior, unsatisfactory academic performance, low well-being, and so forth [2–6]. Given the significance of good self-control to individual development and societal harmony, scholars have been striving to understand the factors that account for individual differences in self-control, because exploring this issue may shed light on the design and implementation of palliative promotion programs aiming to enhance individual self-control ability.

Existing studies have identified a number of genetic and environmental (e.g., parenting, school, neighborhood, and peers) factors that distinguish those who possess good self-control and those who do not [7–11], which suggests that individual self-control is shaped by both genes and socialization. Social norms and morality are guidance driving individuals to act in a socially approved way [12–14], implying that in some sense social norms and morality are sources of self-control. However, individuals acquire social norms and morality not in a vacuum but in a given cultural context, as some cultures may emphasize certain values that particularly facilitate one’s development and recruitment of self-control. In this sense, culture is supposed to associate with the development of self-control. However, the extent to which *culture* relates to self-control has been relatively overlooked, and thus addressing this issue would provide complementary knowledge about the understandings of individual differences in self-control.

Culture consists of many dimensions and individualism-collectivism is one dimension that has been frequently used to examine how culture is related to individual psychological functioning [15]. Individualism-collectivism can be understood at both country- and individual-level levels. In fact, scholars have long assumed that collectivistics possess more self-control than do individualistics, and some studies have examined this assumption based on country-level individualism-collectivism by directly comparing self-control and related constructs across countries [16–18]. However, existing findings are inconsistent, with some studies revealing that collectivistics have higher self-control than individualistics while other studies revealing the opposite. Since country-level individualism-collectivism is not entirely equal to individual-level individualism-collectivism (see below for details), examining the aforesaid assumption based on both country-level and individual-level individualism-collectivism may provide a fresh perspective. However, to date scant research has exerted such an endeavor. Therefore, this study aimed to explore the relationship between individualism-collectivism and self-control in order to provide a greater understanding to this issue.

### Country-level and individual-level individualism-collectivism

Country-level individualism-collectivism is based on Hofstede’s model [19] and reflects how individualistic or collectivistic a nation is. According to this model, individualism and collectivism are seen as two polar on a unitary dimension, with one pole being individualism and the other being collectivism. In other words, high score on one end (e.g., individualism) means low score on the other (e.g., collectivism). Hofstede has created an individualism index (https://www.hofstede-insights.com/product/compare-countries/) to indicate each country’s levels of individualism, with a higher score meaning the country is more individualistic (i.e., less collectivistic). For instance, China has an individualism score of 20 and is often seen as a classic collectivistic culture whereas U.S. has an individualism score of 91 and is usually deemed as a representative of individualistic culture. It is vital to note that country-level individualism-collectivism is not fixed but changes over time. Specifically, when a country’s socioeconomic status grows, the country becomes more individualistic [20]. In this sense, individualism and collectivism may also differ across regions in the same country with huge differences in socioeconomic development [21]. For instance, within the same country well-developed regions are likely to be more individualistic than under-developed regions.

Individual-level individualism-collectivism (also coined as idiocentrism-allocentrism) reflects how individualistic or collectivistic a person is, such that some people can be more individualistic and/or collectivistic than others. Individualism and collectivism at individual level is largely based on Triandis’s model [22]. This model suggests that collectivism and individualism should be seen as two rather than one dimension and that an individual can endorse individualistic and collectivistic values at the same time, depending on the situation [23]. Moreover, Triandis and Gelfand [24] further categorized individual-level collectivism and individualism into four prototypes by considering the levels of equality / hierarchy, namely horizontal individualism, horizontal collectivism, vertical individualism, and vertical collectivism. There are a number of measures assessing individual-level individualism and collectivism, and Triandis and Gelfand’s [24] Horizontal and Vertical Individualism-Collectivism measure is one of the most recognized scales to tap this construct. For instance, this measure assesses four types of individualistic / collectivistic orientation, but it can be summed into two dimensions when necessary to represent a single dimension of individualism and a single dimension of collectivism [25, 26]. Unlike country-level individualism-collectivism, individual-level individual-collectivism is a relatively stable personal value orientation that can affect individuals’ thoughts, feelings, and behaviors [23].

In general, people in collectivistic cultures are likely to be collectivistics and people in individualistic cultures are likely to be individualistics [23]. However, it is crucial to note that such mapping is only probabilistic and not absolute. In other words, not all people in individualistic cultures are individualistics, nor all people in collectivistic cultures are collectivistics. Instead, some people in individualistic cultures can be collectivistics; likewise, some people in collectivistic cultures can be individualistics. Because such culture-individual mapping is only probabilistic, studies that claim collectivistics possess higher levels of self-control than their individualistic counterparts based on a direct cross-national comparison often obtain inconsistent findings. One simple reason is that it is possible that people sampled from a collectivistic / individualistic country in one study can be more collectivistic / individualistic than the ones sampled in another study, even from the same country or region. Hence, scholars have recommended that when investigating the association between individualism-collectivism and one’s psychological functioning, a direct assessment of one’s individualism-collectivism rather than a conjecture of a country’s individualism-collectivism is desirable [15].

### Individualism-collectivism and self-control

The ecological model of human development suggests that multiple systems influence individual’s developmental outcomes and culture is a typical example of the macro system which affects one’s development through other systems [27]. For instance, in a certain cultural context, parents adopt parenting behavior that conforms to the requirements of that culture, which in turn shapes their children’s self-construal and subsequent personality and behavior [28]. Under parents’ supervision and socialization, children establish a sense of their own self, their relationship with others and learn to value both independence and interdependence, and finally internalize the rules about how to behave themselves in the social environment they live in [29]. The effect of cultural orientation on the development of self-control is likely to be robust because self-control is important to fulfill the requirements of that culture [29].

After children have developed their own sense of self-construal, this (relatively) stable individual difference in cultural orientation guides their thoughts, emotion, and behavior. According to Triandis [23], individualistics are autonomous and independent from their in-groups, prioritize personal over in-groups goals, behave primarily based on their attitudes rather than norms of in-groups, and emphasize competition and self-reliance. By contrast, collectivistics are interdependent within in-groups, give priority to the goals of in-groups over their personal goals, behave primarily based on norms of in-groups, and stress harmony relationship within in-groups. To maintain collective good and harmony relationship within the in-group, collectivistics have been taught to exercise self-control to inhibit self-interests and personal desires and to control emotions (particularly the negative ones) since at young age. Thus, collectivistics are considered to be more motivated to exercise self-control on a daily basis than individualistics [30]. The frequent exercise of self-control eventually builds up an individual’s self-control strength in the long run [31]. Daily exercise of self-control may also form a habit of controlling oneself without voluntary effort, which is recently known as an effortless process that fosters and maintains self-control ability [32]. Taken together, in this sense, collectivistic orientation should be positively related to self-control; by contrast, individualistic orientation is supposed to be negatively related to self-control.

Previous cross-national studies have compared self-control and related constructs across different countries, but findings are mixed. For instance, toddlers raised in China and South Korea (usually seen as collectivistic cultures) are found to perform better on behavioral measures of self-control compared to those brought up in Australia and Italy (usually seen as individualistic cultures) [18]. Study also revealed that adults from collectivistic culture (e.g., China) are less sensitive to self-control resources depletion [30] and engage in less impulsive buying behavior [17] than those from individualistic culture (e.g., U.S.). Study based on one cultural context also revealed that individual-level individualistic and collectivistic orientation was negatively and positively related to interpersonal self-control, respectively [33]. However, some research [16] found that Chinese adolescents reported as much trait self-control as their Italian counterparts.

These inconsistent findings may be due to both theoretical and methodological reasons. Theoretically, as mentioned above, country-level individualism-collectivism is not entirely mapped onto individual-level individualism-collectivism. Therefore, a direct cross-cultural comparison should not be seen equal to the relation between individual-level individualism-collectivism and self-control. A good solution to this question is to associate individual-level individualism-collectivism with self-control as well. Methodologically, the captioned studies employed different measures of self-control (e.g., attitude self-control and behavioral self-control measures), which may also yield different outcomes. In this study, we employed both personality scale and behavioral task to assess self-control in order to provide more compelling evidence for this issue.

### Assessment of self-control

Self-control has been widely studied in various branches of psychology (e.g., social psychology, personality psychology, developmental psychology, etc.) and also in other discipline such as criminology. Within this background, a number of analogous terms (e.g., self-restrain, self-discipline, effortful control, self-regulation, etc.) have been proposed, but these terms are considered to be under the same umbrella of voluntary self-governance [34]. Correspondingly, numerous instruments have been developed to assess self-control and these tools can be divided into two broad categories, namely questionnaires and behavioral tasks. Tangney et al.’s Brief Self-Control Scale (BSCS) [1] is one of the most frequently used measures to assess one’s trait/attitudinal self-control whereas the Stroop task is one of the widely employed laboratory tasks to tap individual’s behavioral self-control (i.e., the ability to override dominant response to respond to a subdominant stimulus). A recent meta-analysis which summarized the convergent validity of different self-control measures found that the association between self-report informant self-control questionnaires and behavioral tasks is modest [34]. Hence, scholars have recommended that it is desirable to include both attitudinal and behavioral measures of self-control to have a better view of the construct [34]. Following this suggestion, we employed both the BSCS and Stroop task to assess participants’ self-control in this study.

### The present research

This study explored the relation between individualism-collectivism with self-control. To this end, we tested this association among two samples from countries seen as representative of collectivistic (e.g., China) and individualistic (e.g., U.S.) cultures. As mentioned above, both attitudinal measure (i.e., the BSCS) and laboratory task (i.e., computer-based Stroop task) were used to measure self-control. Besides, scholars have considered that individualistic orientation should be more relevant in individualistic countries whereas collectivistic values are more endorsed in collectivistic countries [35, 36], we also explored whether the associations between individual-level individualism and collectivism with attitudinal and behavioral self-control differed in China and U.S. by examining the interaction effect between country-level and individual-level individualism-collectivism. Gender, age, and religion status have been found to affect self-control [37–39] and thus were controlled as covariates in the regression models.

## Materials and methods

### Participants and procedure

Two samples were recruited. Chinese undergraduates were recruited from a large University in a developed metropolitan city located in Southern China (*N* = 542; 211 males, 327 females, 4 missing; M_age_ = 19.07 years, SD = .98). U.S. undergraduates were recruited from a large University in the Southeast (*N* = 446; 107 males, 339 females M_age_ = 18.82 years, SD = 1.68). All participants undertook the study either for a lottery drawing or in exchange of extra course credits.

The IRB of the University of Kentucky (U.S.) and Guangzhou University (China) approved the current study. Signed consent were obtained before data collection. The study was administered in a research lab in both countries. One difference in the administration procedure was that Chinese participants answered the questionnaires in paper-and-pencil format whereas U.S. students did so online. All participants were asked to work on the Stroop task before completing the survey. All materials were presented in the native language of the respective country.

### Measures

#### Individual-level individualism-collectivism

The Horizontal and Vertical Individualism and Collectivism Scale (HVICS) [24] was used to assess individual-level horizontal individualism, horizontal collectivism, vertical individualism, and vertical collectivism. This questionnaire was developed based on U.S. samples but has been validated and used in Chinese samples, showing sufficient psychometric properties [25, 26]. Measurement invariance tests were conducted in Mplus 7.31 to examine whether this measure was equivalent between the two samples. Measurement invariance was tenable if the decrease of the value of CFI was no greater than 0.01 while other fit indices such as RMSEA (< 0.08) and CFI (> 0.90) were acceptable [40]. The results revealed that configural invariance was roughly supported, *χ*^2^ (177) = 640.14, RMSEA = 0.073, CFI = 0.873. Metric invariance was not supported (*χ*^2^ (189) = 720.58, RMSEA = 0.076, CFI = 0.854), since the changes in the value of CFI was larger than the cut-off point (i.e., 0.01). After scrutinizing the results, we found that releasing factor loading of three items might increase the model fit. Based on this, we re-examined the model fit after setting the factor loadings of three items freely estimated. The results supported partial invariance of the scale, *χ*^2^ (186) = 668,71, RMSEA = 0.073, CFI = 0.867, since the decrease of the value of CFI less than 0.01. Thus, we assumed that this measure was roughly equivalent between the two samples. In the present study, following prior studies’ suggestion [25, 26], horizontal and vertical individualism were combined into a single *individualism* dimension and horizontal and vertical collectivism were combined into a single *collectivism* dimension in order to provide a more concise understanding of the individualism and collectivism on self-control. The scale consists of 16 items rated on a nine-point Likert scale (“1 = absolutely disagree” to “9 = absolutely agree”). A higher score of individualism indicates participants were more individualistic whereas a higher score of collectivism represents participants were more collectivistic. Sample items were “Parents and children must stay together as much as possible” (collectivism) and “winning is everything” (individualism).

#### Attitudinal self-control

Tangney et al.’s Brief Self-Control Scale (BSCS) [1] was used to assess participants’ perception of their own self-control ability. This scale has been validated and used in Chinese samples, showing good psychometric properties [41]. Measurement tests were conducted to examine whether this scale was equivalent between the two samples. The results showed that configural invariance was supported, *χ*^2^ (112) = 287.48, RMSEA = 0.058, CFI = 0.922. Metric invariance was not supported (*χ*^2^ (124) = 332.78, RMSEA = 0.060, CFI = 0.907), since the changes in the value of CFI was larger than 0.01. After inspecting the results, we found that releasing two items might increase the model fit. Based on this, we re-examined the model fit after setting the factor loadings of two items freely estimated. The results supported partial invariance of the scale, *χ*^2^ (122) = 306.272, RMSEA = 0.057, CFI = 0.918, since the decrease of the value of CFI less than 0.01. In sum, we considered that the BSCS was invariant between the two samples. This scale has 13 items rated on a five-point scale (“1 = not like me at all”, “5 = like me very much”), with a higher score indicating better self-control ability. Sample items are “I am good at resisting temptation” and “Sometimes I can’t stop myself from doing something, even if I know it is wrong”.

#### Behavioral self-control

**Fig 1:**
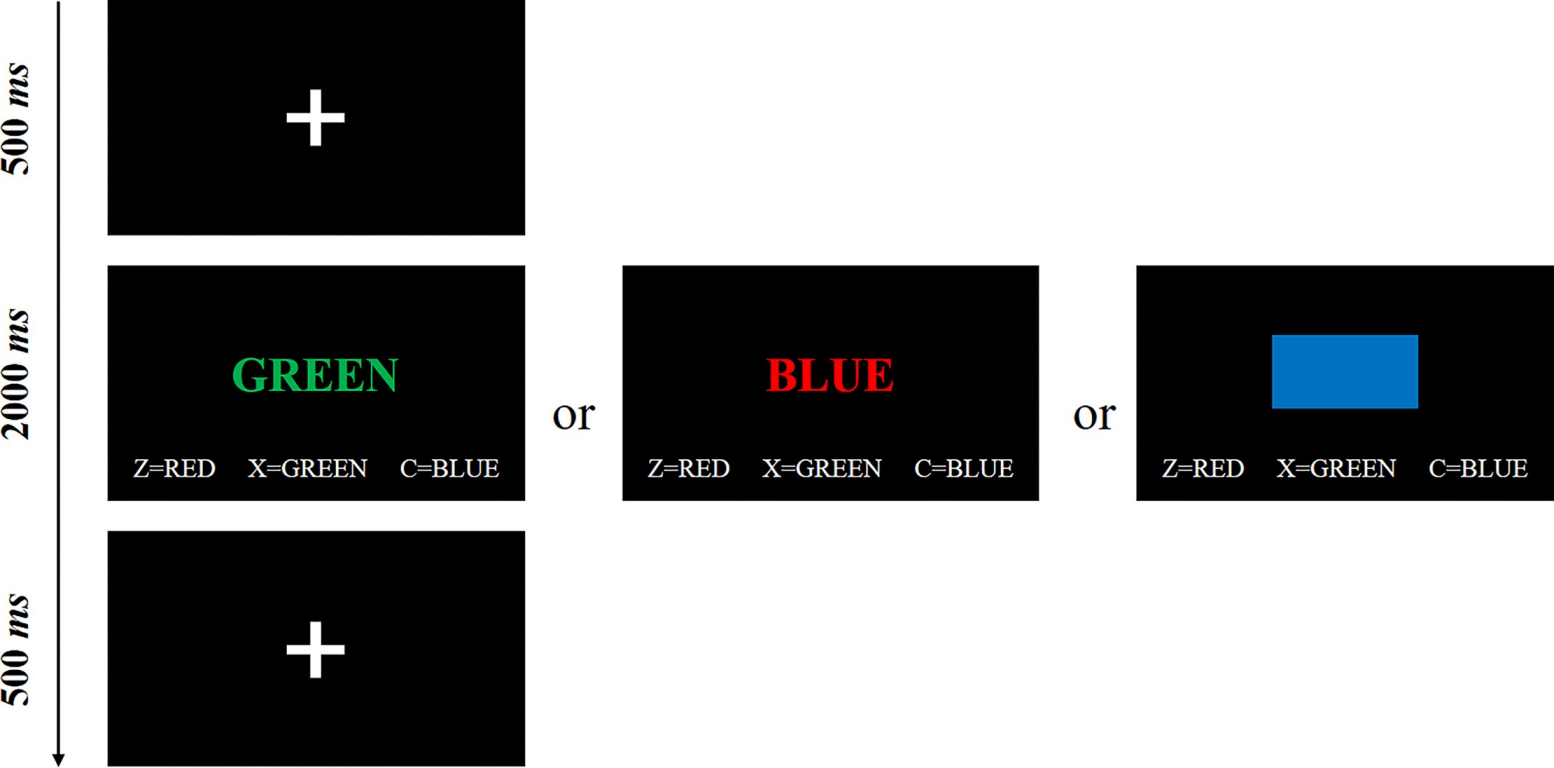
Illustration of Stroop task.

A computer-based word-color Stroop task was developed with E-Prime 2.0 to assess participants’ behavioral self-control, following the version used by Job and colleagues who used Stroop inference effect as the indicator of behavior self-control [42]. The current task consisted of 40 congruent, 40 incongruent, and 40 neutral (a rectangle) trials. All stimuli (i.e., a word or a rectangle in different color) were presented in a 22-inch monitor in a laboratory that accommodated 6 to 8 participants at a time. At the beginning of each trial, there was a 500ms “+”. Then, a stimulus was presented and participants were requested to respond to the color (i.e., red, green or blue) of the stimulus within 2 seconds as fast and accurately as possible by pressing corresponding buttons on the QWERT keyboard. The procedure is illustrated in Fig 1. Following prior research [42], the Stroop interference effect was calculated based on correct trials by subtracting the reaction time (RT) of congruent from incongruent trials (i.e., RT _incongruent trials_−RT _congruent trials_). A higher value indicates participants spend more time inhibiting dominant responses of naming the meaning of the font (higher inner conflict) and thus suggests *lower* self-control. A total of 49 participants (27 Chinese and 22 U.S., about 5% of the full sample size) did not complete the task and thus were excluded from the analyses regarding the Stroop task. Of the remaining 939 participants, 17 participants had outliers (> ± 3 SD) in the congruent and/or incongruent trials and therefore were not included in the analyses (about 1.2% of the full sample size), as suggested by prior research [42, 43]. Hence, Stroop interference scores obtained from 510 Chinese (94.1% of the original Chinese sample) and 412 U.S. (92.4% of the original U.S. sample) participants were included in the final data analyses.

### Data analytic plan

Data were analyzed in SPSS with .05 as conventional level of significance. First, descriptive statistics were carried out to understand the central tendency of each variable. Second, Pearson correlation analyses were conducted to capture the bivariate association between country-level individualism-collectivism, individual-level individualism, individual-level collectivism, and attitudinal and behavioral self-control. Finally, in order to explore the association between individual-level individualism-collectivism with attitudinal and behavioral self-control and whether these relations differed in terms of magnitude and/or direction in the two samples, hierarchical regression models were implemented based on *Z* scores. In this model, covariates, country, individual-level individualism and collectivism were transformed into standardized score and the interaction terms between country-level and individual-level individualism-collectivism were calculated. These variables were entered in the regression model separately, controlling for gender, age, and religion. Analyses were conducted separately for attitudinal and behavioral self-control.

## Results

### Descriptive statistics and bivariate correlations

Means, standard deviation, and Cronbach’s alphas are presented in Table 1 and bivariate correlations are displayed in Table 2. As shown in Table 2, country was positive related to attitudinal self-control, such that U.S. participants reported higher attitudinal self-control than did their Chinese counterparts. With respect to individual-level individualism and collectivism, results showed that individualism and collectivism was negatively and positively related to attitudinal self-control, respectively. Moreover, country was positively related to behavioral self-control, suggesting Chinese participants showed better behavioral self-control than U.S. participants. Individual-level collectivism was negatively associated with behavioral self-control but the one between individual-level individualism and behavioral self-control was not significant. This indicated that individuals’ collectivistic orientation was related to better behavioral self-control.

**Table 1:** Descriptive statistics of individual-level individualism-collectivism, and attitudinal and behavioral self-control.

	China				U.S.			
	*N*	Mean	SD	*α*	*N*	Mean	SD	*α*
1.→ Individual-level individualism	542	6.26	.94	.69	446	6.15	1.01	.71
2.→ Individual-level collectivism	542	6.75	.91	.73	446	6.72	.96	.77
3.→ Attitudinal self-control	542	2.94	.60	.77	446	3.13	.66	.84
4.→Stroop interference	510	96.34	90.18	-	412	110.43	89.22	-

**Table 2:** Bivariate correlation between individual-level individualism and collectivism with attitudinal and behavioral self-control.

	Attitudinal self-control	Behavioral self-control
Country (0 = China, 1 = U.S.)	.150**	.078*
Individual-level individualism	-.089**	-.034
Individual-level collectivism	.111**	-.082*

Note

** *p* < .01

* *p* < .05.

### Regression of attitudinal and behavioral self-control on individualism and collectivism

Hierarchical regression was conducted and results are summarized in Table 3. With respect to attitudinal self-control, after controlling for demographic variables, country was positively related attitudinal self-control (*B* = .171, *p* < .001), suggesting U.S. participants reported higher levels of attitudinal self-control than Chinese participants. Beyond this, individual-level individualism was negatively related to attitudinal self-control (*B* = -.128, *p* < .001) whereas individual-level collectivism was positively associated with attitudinal self-control (*B* = .153, *p* < .001). No significant interaction effect was found.

**Table 3:** Regression of attitudinal and behavioral self-control on individualism and collectivism.

	Attitudinal self-control					Behavioral self-control				
	B	S.E.	*p*	*R*^2^	Δ*R*^2^	B	S.E.	*p*	*R*^2^	Δ*R*^2^
**Step 1**				.007	.007				.006	.006
Gender	-.0164	.033	.629			.041	.031	.182		
Age	-.037	.033	.258			-.034	.035	.329		
Religion	-.034	.036	.342			-.048	.034	.159		
**Step 2**				.028	.020***				.012	.006*
Country	.171	.036	< .001			.076	.034	.027		
**Step 3**				.056	.029***				.018	.006^†^
Individual-level Individualism	-.128	.034	< .001			.002	.031	.941		
Individual-level Collectivism	.153	.033	< .001			-.074	.031	.016		
**Step 4**				.057	.001				.020	.001
Individual-level Individualism × country	.020	.033	.539			-.019	.031	.529		
Individual-level Collectivism × country	.013	.033	.683			.031	.030	.305		

Note

*** *p* < .001

** *p* < .01

* *p* < .05

† *p* = .061; country coded 0 = China, 1 = U.S.

religion coded 0 = no religion, 1 = has religion.

For behavioral self-control, after controlling for demographic variables, country was positively related to behavioral self-control (*B* = .076, *p* = .027), suggesting Chinese participants had higher behavioral self-control than did U.S. participants. Beyond this, individual-level individualism was not significantly related to behavioral self-control (*B* = .002, *p* = .941) whereas individual-level collectivism was negatively related to behavioral self-control at statistically significant level (*B* = -.074, *p* = .016). This indicated that individual-level collectivism was related to better behavioral self-control. No significant interaction effect was found.

## Discussion

This study sought to examine the association between individualism and collectivism with self-control using both attitudinal and behavioral measures. Our findings revealed that (1) Chinese participants reported less attitudinal but showed more behavioral self-control than their U.S. counterparts; and (2) individual-level individualism and collectivism was negatively and positively related to attitudinal self-control, respectively, and individual-level collectivism was related to higher levels of behavioral self-control.

Collectivistics have been assumed to possess more self-control than individualistics, but prior cross-cultural studies yield mixed findings [16–18, 30]. Similarly, our current results based on country-level individualism-collectivism also found mixed support for this assumption. To be specific, this assumption was supported when self-control was measured by behavioral but not by attitudinal measure. One plausible reason may be because of the differences in proneness of self-enhancement between North American and Eastern individuals [44, 45]. Specifically, although Easterners make favorable self-evaluations, it is not as prevalent and explicit as U.S. individuals [46, 47]. In the current study, U.S. participants might rate themselves to have higher self-control to give others a good impression, but this self-enhancement strategy is not usually used in Chinese context. However, when it comes to behavioral self-control, Chinese participants indeed showed higher self-control than U.S. participants, which suggests that Chinese participants seem to be more adept at enduring the cognitive burden of the Stroop task and showing consistent good performance over the task than U.S. participants. This may reflect Chinese participants’ long-term exercise of inhibition / suppression ability.

From the perspective of individual-level individualism–collectivism, our findings lent consistent support to the aforesaid assumption. Specifically, we found that individual-level collectivism was related to higher levels of both attitudinal and behavioral self-control, despite that we do not have concrete explanations about why individual-level individualism was significantly related to attitudinal but not to behavioral self-control. Moreover, no interaction effect between country and individual-level individualism and collectivism was found. This suggests that the association between individual-level individualism and collectivism with attitudinal and behavioral self-control did not differ in terms of magnitude or direction, and these findings can be applied to both China and U.S. samples. Such results are in consistent with recent research which discloses that family allocentrism, one’s allocentric orientation towards family, was related to better (attitudinal) self-control in both Chinese and Italian adolescents with equal magnitude [48]. Taken together, the long-held assumption that “collectivistics have more self-control than individualistics” seems to enjoy more consistent support when individual-level individualism-collectivism framework is used.

Self-control is a complex constructs that encompasses multiple aspects, and thus scholars have called for multiple modalities to assess it [34]. In this study, we used both self-report questionnaire and behavioral task to assess self-control. Although a growing stream of evidence suggests that performance on cognitive measures of inhibition do not reliably correlate with attitudinal self-control or related constructs [49, 50], prior meta-analysis [34] reveals that the correlation between attitudinal self-control and behavioral measures is modest and significant. In addition, our results showed individualism-collectivism affect both one’s perception (i.e., attitudinal) and their exertion (i.e., behavioral) of self-control. These evidences suggest that future research which examines the relationship between cultural orientation and self-control should rely on both attitudinal and behavioral measures to assess self-control. Including behavioral self-control not only provides triangulation of the results, but it may also help reduce the problem of self-enhancement.

As scant research has examined the association between individual-level individualism and collectivism with self-control, future study may expand the research scope in several aspects. First, this study only focused on samples from two countries, and thus it would be premature to draw any firm conclusion. Future research may examine this issue in more countries and then apply multi-level analysis to examine the influence of country- and individual-level individualism-collectivism on self-control in a more sophisticated manner. Second, another promising venue is to examine whether manipulation of one’s momentary cultural orientation with cognitive priming technique could change one’s self-control ability. Third, one more possible research line is to employ longitudinal research design to investigate the dynamic relationship between individualism-collectivism orientation and the development of self-control. Fourth, culture consists of many dimensions and individualism-collectivism is only one of them. Recent work by Vignoles and colleagues (2016) identified seven dimensions to describe cultural differences in selfhood [21]. Thus, a future avenue regarding this issue is to examine other cultural dimensions (e.g., tightness-looseness, power distance, etc.) in relation to self-control. Finally, self-control operates in different steps, including self-monitoring, comparing the current state with standards, and regulating the discrepancy between the current state and standards [51]. Self-control examined in this study is more about one’s regulatory ability. Future research may also examine how cultural variables affect individuals’ acquisition of social norms and the development of self-monitoring processes.

This study is not without limitations. One drawback is the results are only based on undergraduates recruited in one city, which limits its generalizability to other regions or age groups. Replicating this study in other age groups is important for two reasons. On the one hand, it is considered that the university subcultures in China and U.S. are similar in the contemporary period of cultural globalization [52]. Examining this issue among participants other than university students may draw an even more complete picture of the relationship between culture and self-control. On the other hand, as mentioned in the introduction, individuals develop their self-construal through parents’ socialization since they are young. Examining this topic among younger participants may better disclose the trajectory of how culture affects one’s development of self-control. In addition, we could not rule out the influence of different patterns of administration, although research has supported that pencil-and-paper and online surveys generally do not differ in psychometric properties [53, 54]. Another minor limitation we must acknowledge is we did not check whether the computer parameters were entirely identical between China and U.S. although the monitor size was the same. Nevertheless, this study provides fresh insights about the association between individualism-collectivism with self-control. In conclusion, our data show that both country-level and individual-level individualism-collectivism have some influences on individual differences in self-control, implicating the utility of culture in explaining the individual differences in attitudinal and behavioral self-control.

## Supporting information

S1 File Culture and self-control—Minimized data. (SAV)
